# Special Meeting of the Hospital Saturday Fund

**Published:** 1911-07-22

**Authors:** 


### SPECIAL MEETING OF THE HOSPITAL SATURDAY FUND.

### ITS PROSPECTS UNDER THE INSURANCE BILL.

A special meeting of the Board of Delegates of the Hospital Saturday Fund Association was held on Wednesday evening, July 12, at the offices, Gray's Inn Road, for the purpose of receiving and considering the report of the Executive Committee as to the provisions of the National Insurance Bill and their probable effect upon the work of the Fund. Sir Savile B. Crossley, Bart. (the chairman of the Fund), presided. There were 111 delegates present, including a considerable number of representatives of the participating medical charities.

The report was presented by Mr. W. F. Chalkley, the chairman of the Council.

Sir Savile Crossley said he thought the report as drawn up by the Executive was an exceeding good one. He had read it carefully, and thought there was very little to find fault with. There were one or two objections in the report as then drafted, and he intended to put them forward. The report stated that the Hospital Saturday Fund was in favour of the Bill, whereas what he desired to say was that they were in favour of the intentions and purposes of the Bill as a whole. (Hear, hear.) He objected to the statement that they were in favour of the Bill, because they did not know yet what the Bill is, and those who had had experience in Parliamentary life would know that any Bill as originally drafted was liable to very serious alteration. Serious alterations had been made in many other Bills lately before the House of Commons. The Chairman having put before the meeting the various "objections" in the Executive Committee's report, these were met by amendments, and an altered statement was substituted, after nearly two hours' discussion, for the original report. It was resolved that a copy should be sent to the Chancellor of the Exchequer, with the request that he would receive a deputation from the Hospital Saturday Fund Association in respect to the matter.

Mr. W. F. Chalkley expressed the opinion that Friendly Society men who are connected with the approved Societies would benefit eventually under the Bill. They might, for some time, be called upon to pay 4d. per week to their employer—say six months at the outside—but that was a question for the Society to arrange themselves. After that period all the Friendly Societies could relieve their members of that amount of contribution, and then, under the clause of the Bill if carried on the lines suggested, they would be able to give their members greater benefits. He had no reason to suppose that any Friendly Society man, at any rate, after the first few months of the operation of the Bill, would fall off with his work in connection with the Hospital Saturday Fund. He was almost certain in his own mind that Friendly Society men, as a body, were not so selfish—(hear, hear)—in their work as to say, we decline, for the sake of our wives and families, to contribute a penny a week toward the work of the Hospital Saturday Fund; therefore he did not think they needed any sympathetic feeling from that meeting. They were prepared, when the time came, to fight their own battle, and he was quite sure that they would still help the work of the Hospital Saturday Fund. (Cheers, and "Hear, hear.")

Mr. R. B. D. Acland, K.C. (vice-president of the Fund), thought it was reasonable that the Societies who would receive benefits for their contributions should make some contribution to the hospitals in a way that they would leave to the Chancellor of the Exchequer. He strongly deprecated the destruction of the voluntary system, and would have them leave nothing in the proposals which would enable the Chancellor of the Exchequer to put the Treasury on to the track of the hospitals; if they did, he was sure that the voluntary system of hospitals would be damned for ever. (Hear, hear.)

Mr. D. F. Pennant (hon. secretary, Queen's Jubilee Institute for Nurses) contrasted the position of the District Nursing Association with that of voluntary hospitals in respect to the effect that the Bill would have upon them. He hoped that the result of the Bill might be an alliance between the Societies which are in existence and those which will come into existence under the Bill. He thought it was very reasonable that hospitals should be paid for work done for people insured under the Bill.

A vote of thanks to the Chairman concluded the proceedings.

